# Effect of Mechanical Shaking on the Physicochemical Properties of Aqueous Solutions

**DOI:** 10.3390/ijms21218033

**Published:** 2020-10-28

**Authors:** Sergey V. Gudkov, Nikita V. Penkov, Ilya V. Baimler, Gennady A. Lyakhov, Vladimir I. Pustovoy, Alexander V. Simakin, Ruslan M. Sarimov, Ivan A. Scherbakov

**Affiliations:** 1 Prokhorov General Physics Institute of the Russian Academy of Sciences, 119991 Moscow, Russia; ilyabaymler@yandex.ru (I.V.B.); gen.lyakhov@gmail.com (G.A.L.); pustovoy@nsc.gpi.ru (V.I.P.); avsimakin@gmail.com (A.V.S.); rusa@kapella.gpi.ru (R.M.S.); ivan11444@mail.ru (I.A.S.); 2 Institute of Cell Biophysics of the Russian Academy of Sciences, PSCBR RAS, 142290 Pushchino, Russia; nvpenkov@yandex.ru

**Keywords:** water, shaking, reactive oxygen species, molecular oxygen, nanobubbles, long-lived luminescence

## Abstract

Long-lived luminescence in the blue region was found to occur in deionized water saturated with atmospheric gases following mechanical shaking. Luminescence intensity decreased exponentially after the cessation of stress. During vigorous mechanical shaking, we observed gas bubbles in solution, and the liquid–gas interface area increased noticeably. At the same time, the concentration of molecular oxygen decreased, which could not be attributed to the water warming up with exposure to mechanical stress. However, deaerated water rapidly became saturated with gases following mechanical stress. The recommendation that cell culture media should be mixed after they are removed from the fridge in order to allow saturation with oxygen is probably misleading. It was shown that gases existed in water both in the form of individual molecules and nanobubbles. Mechanical stress did not influence the number or size of nanobubbles. While gas nanobubbles were absent in freshly prepared deaerated water, they appeared following exposure to mechanical stress. In addition, in mechanically treated gas-saturated water, there was seemingly an equilibrium shift towards the decomposition of carbonic acid to water and carbon dioxide. At the same time, the pH of water tended to increase immediately after mechanical stress. It was demonstrated that reactive oxygen species (ROS) form in gas-saturated water under mechanical stress (30 Hz, amplitude of 5 mm). The relative generation rate of hydrogen peroxide and of the hydroxyl radical was 1 nM/min and 0.5 nM/min, respectively. It was found that with an increase in the frequency of mechanical action (*f*), the rate of ROS generation increased in proportion to *f ^2^*. The major pathways for hydrogen peroxide generation are probably associated with the formation of singlet oxygen and its further reduction, and the alternative pathway is the formation of hydrogen peroxide as a result of hydroxyl radical recombination.

## 1. Introduction

Exposure of water and aqueous solutions to mechanical stress is used to address a large number of problems in various areas of human activity (particularly in pharmaceutical, food, chemical, and other industries). Mechanical stress is generally applied to aqueous solutions for dissolution and/or mixing and/or wetting processes. There are also more specific applications of mechanical stress, such as particle disaggregation [1] or flotation concentration of molecules [2]. It was discovered that extrinsic molecules present in water can be damaged when they are exposed to vigorous mechanical stresses. For example, in 1938, Henry B. Bull proposed one of the earliest protein denaturation models using mechanical stress [3]. In around the middle of the previous century, such models were introduced in many industries, even in unexpected ones such as woodworking [4]. There were even attempts to use mechanical stress as a stress test for protein quality [5].

Today, changes in the properties of substances and their mixtures are studied within mechanochemistry. A reasonable amount of facts have been collected that suggest that mechanical stress can influence molecules dissolved in water, whereas, according to one viewpoint, water itself, due to its simple composition (only three atoms of two types), does not undergo substantial change [6]. The scientific community has generally been quite skeptical of information regarding changes that occur in water as a result of “weak” non-ionizing exposures, including mechanical stresses. This is basically due to a number of abstract notions used to interpret the effects, e.g., very large water clusters, water crystals, and water memory [7].

Now it has become clear that mechanical stresses can influence not only abstract water structures but also the universally recognized physical properties of water. To note, there have been a great number of experiments performed under extreme conditions (e.g., high pressure, high-frequency mechanical stress, super-critical states) or with the addition of some chemical reagents to water [8].

We have only considered experiments carried out under normal laboratory conditions (atmospheric pressure, room temperature, and subsonic-frequency stresses). In this way, it has been demonstrated that near-UV scattering spectra of water change significantly following succussion, with the changes persisting for a several hours after stress has been applied. [9]. Experiments using dynamic light scattering have shown that mechanically treated deionized water can undergo changes in its physicochemical properties, which persist for over a week [10]. Subsonic-frequency mechanical oscillations have been shown to increase the redox potential of water, which is also maintained for a considerable period of time [11]. When under mechanical stress, water is exposed to the stress-optical effect, which is characteristic of the majority of homogeneous Newtonian fluids [12].

Unfortunately, there are currently no convincing explanations for a number of observed phenomena, as their underlying physicochemical mechanisms have not been sufficiently investigated. This study aims to comprehensively investigate changes that occur in water under mechanical stress. The experiments were designed so that they could allow summarizing and substantially explaining the known facts.

## 2. Results

**Figure 1:**
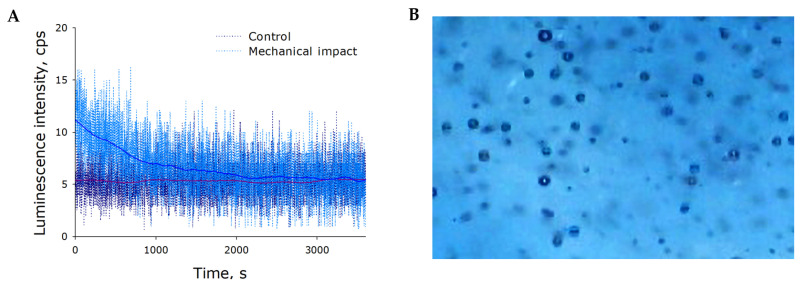
The effect of mechanical stress (30 Hz, amplitude of 5 mm, for 5 min) on intrinsic water luminescence (**A**) and gaseous phase distribution (**B**). The photograph was taken shortly after the mechanical treatment.

The effect of mechanical stress on the intrinsic luminescence of water was studied (Figure 1). The background luminescence of control water was shown to be around 5 cps. On the whole, the luminescence intensity of intact water was not significantly changed for at least 1 h. Mechanical treatment of water (30 Hz, amplitude of 5 mm, for 5 min) at the beginning of the measurement led to a twofold increase in luminescence intensity compared to the control. The luminescence of mechanically treated water attenuated exponentially with time and equated with the luminescence of control samples after 30–40 min. Interestingly, the phenomenon of increasing intensity of water luminescence was only observed for gas-saturated water, whereas no such increases were observed for the luminescence of freshly distilled water. Thus, it was shown that water saturated with atmospheric gases begins to luminesce under intense mechanical stress.

What can mechanically induced water luminescence be attributed to? Apparently, luminescence is linked to gases dissolved in water; however, even with this being the case, there can be several alternative hypotheses. Luminescence is usually observed upon relaxation of excited compounds (for gases, it is transition of oxygen from singlet to triplet state), or during cavitation or radical recombination. It is known that the singlet–triplet transition of oxygen is accompanied by red spectrum radiation. Cavitation and radical reactions are characterized by blue-spectrum light emission. Red or blue absorption filters were inserted between water samples and the photomultiplier in some of the experiments. It was shown that shaking-induced water luminescence predominantly occurred in the “blue” range of the spectrum. Therefore, cavitation of nanobubbles in solutions and/or radical reactions in water are the most common sources of luminescence. For a detailed understanding of the nature of luminescence, we made a number of physicochemical measurements.

**Figure 2:**
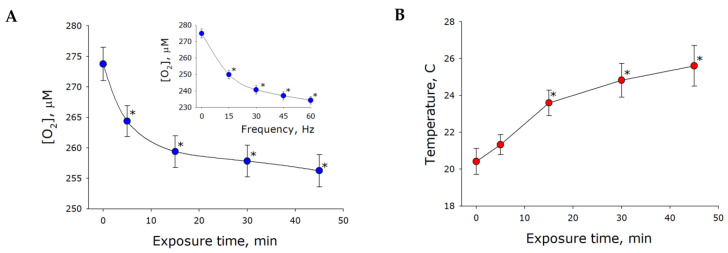
The effect of mechanical stress (30 Hz, amplitude of 5 mm) on molecular oxygen concentration in water (**A**) and water temperature (**B**). The insert to figure (**A**) displays the effect of frequency of mechanical stress on the test parameter (exposure time 5 min). The data are presented as the mean and standard error of the mean for six independent assays. Data differ significantly from control values (0 min) at *p* < 0.05 (*).

Gas bubbles were visible in the solution when it was vigorously shaken (Figure 1B). At this point, the liquid–gas interface area appeared to increase noticeably, which should presumably cause changes in the concentration of gases dissolved in the water. Therefore, the effect of mechanical stress on molecular oxygen concentration in water was studied (Figure 2A). It was shown that the concentration of molecular oxygen dissolved in intact water was approximately 273 µM. It was found that the molecular oxygen concentration was reduced to 264 µM after 5 min exposure to mechanical stress. Molecular oxygen concentration was observed to decrease with increasing exposure time. For example, after 45-min exposure, the concentration of molecular oxygen was down to 257 µM (decrease by 6% relative to control). The rate of oxygen release from water increased with increasing frequency of mechanical stress (Figure 2A, insert). Therefore, was demonstrated that oxygen concentration decreased in water under mechanical stress. To note, the concentration of molecular oxygen recovered to the equilibrium level within a few hours post-exposure.

The movement of water molecules appeared to intensify under mechanical stress, which could result in temperature increases. Increased temperature might in turn account for partial deaeration of the samples. The effect of mechanical stress on water temperature was examined (Figure 2B). The experiments were performed at room temperature (about 22 °C). Water temperature was shown to increase by 1 °C after 5 min of mechanical treatment. It is known that the solubility of molecular oxygen decreases by less than 5 µM with an increase in temperature from 22 °C to 23 °C. Therefore, reduced concentrations of molecular oxygen in water can only partially be explained by temperature changes.

**Figure 3:**
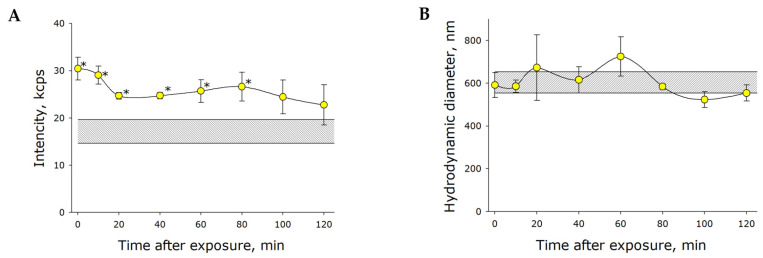
The effect of mechanical stress (30 Hz, 5 mm amplitude) on light scattering intensity (**A**) and hydrodynamic diameter (**B**) of nanosized gas bubbles. The shaded area represents the parameters of the control (gas-saturated water before mechanical treatment). The data are presented as the mean and standard error of the mean for six independent assays. Data differ significantly from control values at *p* < 0.05 (*).

The molecular oxygen concentration measured in water decreased but it was unclear where the molecular oxygen disappeared to. There are at least two alternative hypotheses: (1) gases released from water into the atmosphere, and (2) gases converted into nanosized particles (bubstons), in which gas was not detectable by polarography or by the majority of methods for measuring gas concentration in liquids. Dynamic light scattering was used to examine gas redistribution in bulk water. It was shown that freshly prepared water did not incorporate light scatterers. Light scattering in water increased as it became more saturated with gases. Figure 3A illustrates the effect of mechanical shaking on the intensity of light scattering in water. The intensity was shown to increase by nearly twofold immediately after the application of mechanical stress, remaining at this level for about 2 h post-exposure. At later time points, the scattering intensity restored to control values. Thus, we recorded abrupt increases in the number of gas bubbles immediately after shaking. Visible, large bubbles were observed to rise rapidly to the surface after the cessation of stress. For micrometer sized bubbles, reaching the surface apparently took several hours. Submicron sized bubbles, in which the Archimedes force was canceled out by the liquid’s viscosity, remained within the liquid. We can state that the number of these bubbles did not sufficiently increase after mechanical stress. However, more bubble-phase gas can be stored up not only when the bubbles increase in number, but also when they grow in size. Therefore, the effect of mechanical stress on bubble hydrodynamic diameter was examined (Figure 3B). It was shown that the average bubble diameter was almost unaffected by mechanical stress. Therefore, we showed that deaeration of mechanically treated water was associated with gas release into the atmosphere via forming and rising bubbles.

**Figure 4:**
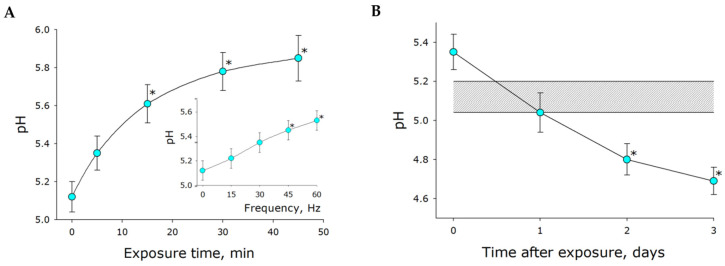
The effect of mechanical stress (30 Hz, 5 mm amplitude) on water pH. (**A**) The effect of exposure time on pH. The insert displays the effect of frequency of mechanical stress on the test parameter (amplitude of 5 mm, exposure time of 5 min). The measurements were made immediately after stress exposure. (**B**) Changes of pH observed for several days post-exposure. The shaded area represents the parameters of water before mechanical treatment (control). The data are presented as the mean and standard error of the mean for six independent experiments. Data differ significantly from control values at *p* < 0.05 (*).

Molecular oxygen was shown to release from water under mechanical stress. We suggested that carbon dioxide and carbonic acid could also release from mechanically treated water. This process should influence water pH. The effect of mechanical stress on water pH was studied (Figure 4A). The measurements were made immediately after stress exposure. The determined pH of intact water was about 5.1. Water pH tended to increase with increasing exposure time. Increasing frequency of mechanical stress resulted in increased rate of pH change (Figure 4A, insert). It is widely recognized that water has a pH of 7 under perfect conditions. Real-life water has lower pH values due to saturation with carbon dioxide. These findings indirectly point to carbon dioxide also releasing from liquids under mechanical stress. After stress exposure, the system gradually restored equilibrium and the pH was observed to decrease as a result of carbon dioxide dissolution. No such effect was observed when the atmosphere above the vessel was changed to argon. Interestingly, the equilibrium pH value was approximately 0.4 units lower following mechanical exposure than in the pre-exposure steady state (Figure 4).

**Figure 5:**
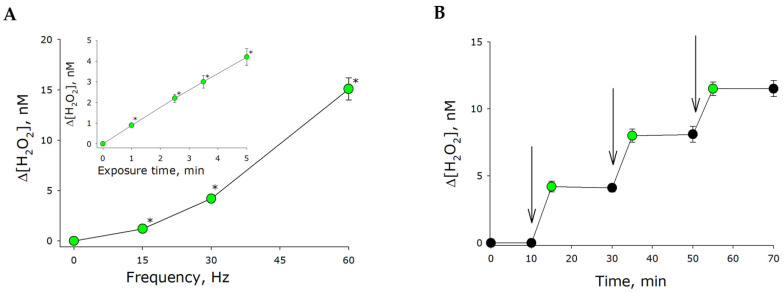
The effect of mechanical stress on hydrogen peroxide concentration in water. (**A**) The effect of frequency of mechanical stress on hydrogen peroxide concentration (amplitude of 5 mm, exposure time of 5 min). The insert displays the effect of exposure time on hydrogen peroxide concentration (30 Hz, amplitude of 5 mm). (**B**) Changes in hydrogen peroxide concentration following multiple mechanical stresses. The time of a single exposure was 5 min, with the frequency and amplitude of 30 Hz and 5 mm, respectively. The intervals during which mechanical stress was applied are indicated with vertical errors. The data are presented as the mean and standard error of the mean for six independent assays. Data differ significantly from control values at *p* < 0.05 (*).

The effect of mechanical stress on electrical conductivity was examined. It was shown that the electrical conductivity of intact water transferred to our vials was about 1 μS/cm. The electrical conductivity of water increased by almost eight times to 7.8 μS/cm after 5 min of mechanical treatment. Electrical conductivity was also observed to increase with increasing exposure time. Of note, increases in water conductivity were observed both immediately after stress and for several days post-exposure. In essence, increasing water conductivity may indicate the generation of charge carriers in the water. Carbonic acid, which disassociates in water, is usually considered to be a charge carrier. However, we can state that the concentration of carbonic acid in water decreased immediately after stress exposure. Therefore, the dissolution of carbon dioxide in water significantly accounted for increases in water conductivity at later time points and did not explain conductivity increases during exposure. Therefore, why does water conductivity also increase immediately after stress exposure? What molecules can be charge carriers? We hypothesized that reactive oxygen species (ROS) could serve as such carriers. Many ROS are charge carriers. The generation of ROS under mechanical stress could account for increasing electrical conductivity as well as for luminescence persisting after stress exposure. The majority of ROS have short lifetimes. Hydrogen peroxide is the most stable ROS. The effect of mechanical stress on hydrogen peroxide concentration in water was studied (Figure 5A). It is known that concentration of hydrogen peroxide in water has been shown to increase linearly under mechanical stress. After 5 min of stress exposure, the hydrogen peroxide concentration was increased by nearly 4 nM. Therefore, the relative rate of hydrogen peroxide generation was approximately 0.8 nM/min. The generation rate was observed to increase with increasing frequency of mechanical stress (Figure 5A, insert). Therefore, it was shown that hydrogen peroxide was generated in water exposed to mechanical stress.

Additive generation of hydrogen peroxide was demonstrated for multiple stress exposures (Figure 5B). The concentration of hydrogen peroxide was unchanged both before and after mechanical stress. Between mechanical stresses, the samples were exposed for 10 min in the dark at room temperature. Hydrogen peroxide generation only occurred during stress exposure. No additional generation of hydrogen peroxide was observed after the cessation of stress. The concentration of hydrogen peroxide generating as a result of mechanical stress remained largely unchanged for many minutes.

**Figure 6:**
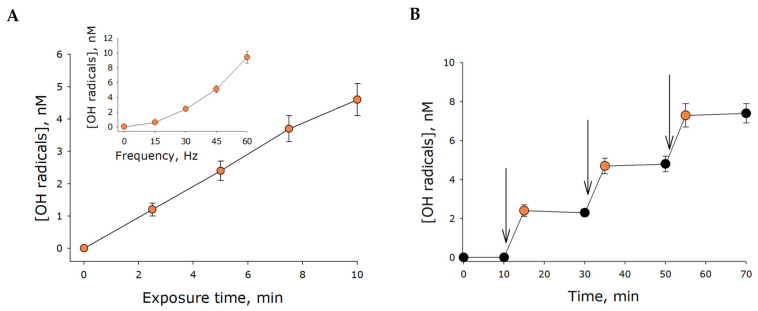
Hydroxyl radical generation in water under mechanical stress (30 Hz, amplitude of 5 mm). (**A**) The effect of exposure time on hydroxyl radical concentration. The insert displays the effect of frequency of mechanical stress on hydroxyl radical concentration (amplitude of 5 mm, exposure time of 5 min). (**B**) Changes in hydrogen peroxide concentration following multiple mechanical stresses. The time of a single exposure was 5 min, with the frequency and amplitude of 30 Hz and 5 mm, respectively. The intervals during which mechanical stress was applied are indicated with vertical errors. The data are presented as the mean and standard error of the mean for six independent assays.

The most reactive ROS is the hydroxyl radical. The effect of mechanical stress on the generation of hydroxyl radicals in water was studied (Figure 6). Hydroxyl radicals were shown to generate in water under mechanical stress. Under mechanical stress, the relative rate of hydroxyl radical generation was approximately 0.5 nM/min. The generation rate of hydroxyl radicals was unchanged for at least 10 min. The generation rate was observed to increase with increasing stress frequency (Figure 6, insert). Thus, it has been shown for the first time that hydroxyl radical generation occurs in water exposed to mechanical stress.

Table 1 shows the effects of different substances on hydrogen peroxide formation. An oxygen effect was detected—the influence of the concentration of water-dissolved oxygen on hydrogen peroxide formation during mechanical treatment. The concentration of hydrogen peroxide generated by mechanical stress in a solution additionally saturated with molecular oxygen to 450 µM was 1.3 times its concentration in control samples. In an argon-saturated solution, wherein the level of molecular oxygen was reduced by one-half to 130 µM, the concentration of generated hydrogen peroxide was 30% lower than in the control. These data indicate that the rate of hydrogen peroxide generation depended on the concentration of molecular oxygen dissolved in the water. It is known that the lifetime of singlet oxygen increases in the presence of heavy water (D_2_O). It was shown that hydrogen peroxide generation was increased by 1.6 times in the presence of 50% D_2_O. Sodium azide, the singlet oxygen quencher, was observed to reduce the H_2_O generation by 30%. These data indicate that singlet oxygen is involved in hydrogen peroxide generation.

By using superoxide dismutase (SOD), it was shown that superoxide anion radicals also participate in hydrogen peroxide generation upon application of mechanical stress. When 10^−3^ units/mL of SOD was added, we observed the concentration of hydrogen peroxide to increase by 60% due to additional dismutation of superoxide anion radicals and hydroperoxide radicals. The addition of tiron, a spin trap for superoxide anion radicals, was observed to reduce the H_2_O_2_ concentration by approximately 60%. After the addition of the non-specific radical scavenger, ethanol, the oxygen release was also decreased by 20%. Contrarily, hydrogen peroxide generation was intensified almost fourfold by the addition of menadione, an electron donor. Therefore, we can argue that hydrogen peroxide generation follows the scenarios described below when under mechanical stress. In the first scenario, molecular oxygen converts from triplet to singlet state and reduces to the superoxide anion radical, which is protonated to the hydroperoxide radical in water. Hydroperoxide radical dismutation leads to the formation of hydrogen peroxide and molecular oxygen, sometimes in singlet state. Since we detected the generation of hydroxyl radicals, we cannot disregard a second pathway for hydrogen peroxide formation that is caused by hydroxyl radical dismutation.

The physicochemical properties of water were observed to change following turbulent mechanical mixing. Would such changes occur upon mixing in low Reynolds number flow? To answer this question, we performed a series of experiments using a microfluidic chip. Laminar-flow mixed water (via Dean vortices), control water, and their mixture were examined. The electrical conductivity and light scattering intensity of water were observed to change as the water was passed over the dual-channel chip in a laminar flow regime (Table 2), similarly to changes in these parameters observed with turbulent mixing. This points to a similar nature of changes occurring in water upon mixing by different methods. A curious observation was when a mixture prepared by adding 1 part of water mixed in flow at low Reynolds number to 99 parts of control water was more similar in its characteristics to the mixed water than to the control water (increased electrical conductivity along with reduced concentration of dissolved molecular oxygen, tendency to increasing pH and light scattering intensity, and somewhat increased hydrogen peroxide concentration). The above means that the principle of additive properties does not apply to components of the mixture and mechanically treated water is able to considerably change the properties of the initial (untreated) water.

In summary, water mixed in flow at low Reynolds numbers exhibited the same trends as water mixed in turbulent flow. The difference was a sufficiently lower (more than by one order of magnitude) rate of change in physicochemical parameters.

## 3. Discussion

It is known that purified water is able to luminesce in the blue–green range when exposed to electromagnetic radiations [13,14]. This type of luminescence is sometimes observed for several hours post-exposure [15]. The physiochemical mechanisms underlying such luminescence have not been completely investigated thus far. It has been suggested that luminescence could develop due to the presence of other impurity molecules, gas molecules (molecular oxygen [16] and/or carbon dioxide [17]), or even organic molecules [18].

Within our study, the presence of organic molecules was unlikely. The development of luminescence (Figure 1) was apparently associated with changes in the gaseous phase (Figure 2, Figure 3 and Figure 4), which led to or influenced ROS generation (Figure 5 and Figure 6). Gases are present in water not only as individual molecules but also in the form of bubbles. It is known that such bubbles can act as seeds for boiling or cavitation [19], i.e., participate in energy transformation from low- to high-density state. In this respect, nanosized gas aggregates (bubstons) are of the most interest [20]. Gas aggregates of this kind can form from different gases [21] and are stable [22]. We have shown that mechanical stress initiates the formation of nanosized gas aggregates in deaerated water and hardly influences already formed bubbles (Figure 3). The phenomenon is probably due to the fact that deaerated water rapidly becomes gas saturated when under mechanical stress, whereas gas concentrations decrease in gas-saturated water (Figure 2). Comparison of the results of this work with the results of [23] allows us to conclude that there is a certain critical concentration of molecular oxygen, below which mechanical action causes an increase in its concentration, and above which it decreases.

Mechanical shaking of aqueous solutions is the basis for experiments in chemistry and biology. In particular, it is a required component for the preparation of solutions with specified concentrations at chemical and microbiological laboratories and is part of production processes of some medicines [24,25,26]. Nowadays, it has been found that the properties of a solution (and therefore presumably its biological activity) can change significantly depending on the mixing method used to mix its components [27]. Our data indicate that these should be taken into account when carrying out some procedures and that theoretical knowledge transfer across scientific fields without a prior detailed experimental study may result in errors. For example, our experiments suggest that the recommendation that cell culture media should be mixed after they have been removed from the fridge to facilitate their saturation with oxygen is misleading. In addition, our results can provide an explanation for many biological effects demonstrated for solutions prepared using mechanical treatment during the dilution process [28,29].

It has been shown that changes in the physicochemical properties of water exposed to mechanical stress depend on its intensity (Figure 2, Figure 4, Figure 5 and Figure 6) and type (in turbulent flow or low Reynolds number flow) (Table 1 and Table 2). It is known that natural waters permanently undergo mechanical mixing and the parameters of such mixing vary within a wide range. Mechanical mixing results in the formation of ROS—extremely reactive compounds. It is known that ROS can, on the one hand, damage biological macromolecules [30] and, on the other hand, play an important signaling and regulatory role in biological systems [31]. ROS are supposed to have had an impact on the development and evolution of life on the earth [32]. It is interesting that the rate of ROS generation is proportional to the frequency squared, that is, the amplitude of the acceleration of the platform of the vibrosystem by the Landau–Lifshitz inertial forces.

Under mechanical stress, hydrogen peroxide generation likely proceeds in two main scenarios. The first one is associated with molecular oxygen activation. It is known that this scenario applies to aqueous solutions exposed to infrared [15], visible radiation [16], and heat [33]. The other scenario is related to hydroxyl anion oxidation. This process was first theoretically predicted over 30 years ago [34] and was superbly demonstrated experimentally in 2019 by using mechanically induced generation of micron sized bubbles in water [35]. To note, this ROS generation scenario ultimately leads to the formation of molecular oxygen as a result of hydrogen peroxide degradation, which could also be of great significance to research into Earth’s oxygen-containing atmosphere.

## 4. Materials and Methods

Water: Deionized Milli-Q water with initial resistivity of 18 MΩ cm at 25 °C was used for the experiments. Freshly prepared deaerated water and water saturated with atmospheric gases for a few days was used. As a bigger part of the work was done using gas-saturated water, it is referred to in the paper as control water.

Turbulent flow mixing of water at high Reynolds numbers (>2000) was carried out using a Multi Reax mechanical laboratory shaker (Heidolph, Schwabach, Germany). The plate of the shaker produced 10 mm amplitude longitudinal vibration at about 15 to 45 Hz, with turbulent mixing, accompanied by intense bubble formation being generated.

For mixing in flow at low Reynolds numbers, we installed an automated microfluidic device consisting of an Atlas syringe pump (Syrris Ltd., Royston, UK), a glass flow microreactor, and a personal computer with a solution preparation algorithm. The two streams were mixed within the microfluidic flow chip by means of Dean vortices forming at the turns of the channel. The chip’s microchannels were 1 mm^2^ in cross-sectional area and 1080 mm long, and the flow rate was 400 µl/min. The water flow ratio of the second channel to the first channel was 1/100. The Reynolds number and the Peclet number were 6.6 and 46.3 in the straight portion of the channel and 35.5 and 250.6 in the curved part, respectively.

Additionally, we examined the role of introducing water mixed in flow at low Reynolds number (a laminar flow with Dean vortices) to control water at about a 1:99 ratio.

We used 20–30 mL vessels in which the pH, conductivity, and other characteristics of deionized water would not significantly change during storage for at least a few hours. The best results were obtained with the use of 20 mL G075Y-27/057-H vials (Glastechnik Gräfenroda GmbH, Gräfenroda, Germany). These vials were used in all the experiments.

Measurement of luminescence: A vial with an irradiated sample was placed in a Biotoks-7AM (NPO ECE, Moscow, Russia) high-sensitivity chemiluminometer operating in the photon counting regime, with a sensitivity band at 380–710 nm. The signal accumulation time and recording interval were 1 s. The photon counting efficiency was about 10%, as determined by calibration against Cherenkov radiation from isotope ^32^P. The luminescence spectral range was estimated using blue or red optical filters with 99% light transmission at 380–520 and 590–800 nm, respectively [15].

Measurement of molecular oxygen: The concentration of dissolved oxygen was measured on an AKPM-1-02 device (REM, Moscow, Russia). The initial oxygen concentration in water was about 270 μM. The saturation of water with argon by bubbling for 20 min led to a decrease in oxygen concentration to 130 μM. The saturation of water with molecular oxygen by bubbling for 20 min increased the oxygen concentration to 450 μM [36].

The temperature was measured using a PTI120 thermal imager (Fluke, Everett, WA, USA) with a field of view of 50° H × 38° V and accuracy of 0.1 °C. Temperature measurements were made immediately after stress exposure. Temperature values were determined by software averaging of captured data.

Dynamic light scattering: The intensity of light scattering and average hydrodynamic diameter (R) of scatterers in water was determined on a Zeta Sizer Nano ZS device (Malvern, Worcestershire, UK). A He–Ne laser with a wavelength of 633 nm was used as a light source. The scattering angle was 173°. The main procedures have been described in detail previously [37].

The pH was measured with an И-500 pH meter (HTK (science and technology complex), Russia). The water was slowly mixed with a magnetic stirrer during measurements [38].

The electrical conductivity was measured with a Cond 6+ conductivity meter (Eutech Instruments, Waltham, MA, USA). A standard measurement protocol was used [38].

Measurement of hydroxyl radicals: A standard measurement protocol was used. Hydroxyl radical levels were determined with a selective sensor for the radicals—coumarin-3-carboxylic acid, whose hydroxylation product, 7-hydroxycoumarin-3-carboxylic acid, fluoresces intensely. The assay sensitivity was <0.1 nM [39].

Measurement of hydrogen peroxide: To quantify hydrogen peroxide in water, we applied the high-sensitivity enhanced chemiluminescence assay using a luminol-4-iodophenol-HRP system [40]. Luminescence intensity was recorded with a Biotoks 7A USE luminescence meter. The test samples were treated physically. The concentration of produced hydrogen peroxide was calculated from calibration curves generated using chemiluminescence intensity measurements for samples spiked with hydrogen peroxide. The initial concentration of calibration hydrogen peroxide was determined by spectrophotometry at a wavelength of 240 nm and molar absorptivity of 43.6 (M-1 × cm^−1^). Prior to measurements, we placed the samples in polypropylene vials (Beckman, Brea, CA, USA), where 0.15 mL of a “count solution” containing 10 mM Tris-HCl buffer (pH 8.5), 50 50 μM p-iodophenol, 50 μM luminol, and 10 nM of horseradish peroxidase was added, with the nanomolar concentrations of H_2_O_2_ determined. The “count solution” was freshly prepared before use. The assay sensitivity allows for the determination of H_2_O_2_ at a concentration of <1 nM [41].

An inhibition assay was used along with the hydrogen peroxide assay. Heavy water was added to test samples (ISOTOPE, Moscow, Russia) in order to increase the lifetime of singlet molecular oxygen [42]. Sodium azide (Dia-m, Moscow, Russia), a physical quencher of singlet molecular oxygen, was added to test samples [43]. To enhance the recombination of superoxide anion radicals, we added superoxide dismutase (Sigma, St. Louis, MO, USA) to test solutions. Tiron (Sigma, St. Louis, MO, USA), a radical trap, was added to test samples in order to detect radical products. Menadione (Sigma, St. Louis, Mo, USA) was used to increase the rate of singlet oxygen reduction. Alpha grade ethanol (Crystal, Moscow, Russia) was used as a non-specific hydroxyl-radical scavenger.

## 5. Conclusions

In sum, we showed that exposure of water to mechanical stress is a complex physical process that leads to changes in water physicochemical properties:

1. Following mechanical treatment, water saturated with atmospheric gases luminesces in the blue range. Luminescence intensity decreased exponentially after the exposure.
2. After mechanical action, the concentration of gases (molecular oxygen and carbon dioxide) in water decreased. In this case, neither the average size nor the number of nanosized gas bubbles changed.
3. We found that hydrogen peroxide and hydroxyl radicals were generated through mechanical action in an aqueous solution, and the concentration of these components quadratically increased with the frequency of mechanical vibrations at constant amplitude.
4. The generation of ROS occurred with mixing in both turbulent and laminar flow with Dean vortices (with a lower intensity), excited in a microfluidic experimental scheme.
5. Important and, possibly, the defining stage of ROS generation under mechanical action was the conversion of molecular oxygen from the triplet to the singlet state.

## Figures and Tables

**Table 1:** The effects of different substances on hydrogen peroxide formation in deionized water following 5-min exposure to mechanical stress (30 Hz, amplitude of 5 mm). The means for three independent assays and their standard errors are provided.

Effect	Δ[O_2_], µM	Δ[H_2_O_2_], nM	K ***
Control	270	4.2 ± 04	1
Ar saturation	130	3.2 ± 0.3	0.7
O_2_ saturation	450	5.4 ± 0.4	1.3
D_2_O (25%)	270	6.8 ± 0.8 **	1.6
Sodium azide (0.1mM) *	270	2.9 ± 0.2 **	0.7
SOD (10^−3^ units/mL)	270	6.9 ± 0.5 **	1.6
Tiron (100 nM)	270	1.6 ± 0.2 **	0.4
Ethanol (0.5 M)	270	3.4 ± 0.3 **	0.8
Menadione (1 µM)	270	16.4 ± 2.3 **	3.9

* The concentration at which sodium azide did not have a noticeable inhibiting effect on peroxidase activity in the “count solution”; ** differs significantly from the control (*p* < 0.05); *** relative change in H_2_O_2_ concentration in the presence of the test agent.

**Table 2:** Changes in the physicochemical parameters of water passed through a microfluidic device under non-mixing or mixing conditions.

Change in the Parameters of Water Passed through a Microfluidic Device	Type of Water Treatment		
	Control	Mixed Water **	Mixture ***
Electrical conductivity, µS/cm	0.91 ± 0.17	1.61 ± 0.11 *	1.74 ± 0.15 *
pH	5.12 ± 0.08	5.17 ± 0.03	5.15 ± 0.06
Light scattering (DLS) intensity, kcps	17.1 ± 2.5	21.9 ± 1.5 *	18.1 ± 1.7
[H_2_O_2_], nM	31.8 ± 2.0	34.2 ± 1.8	33.3 ± 2.1
[O_2_], μM	273.8 ± 2.7	271.9 ± 2.6	267.6 ± 2.5 *

* Differs significantly from the control (*p* < 0.05); ** water passed over the dual-channel chip of the microfluid device; *** mixture prepared by adding microfluid device-processed water to control water (at a 1/99 ratio (*v/v*)).
